# Spatiotemporal Modeling of *Aedes aegypti* Risk: Enhancing Dengue Virus Control through Meteorological and Remote Sensing Data in French Guiana

**DOI:** 10.3390/pathogens13090738

**Published:** 2024-08-29

**Authors:** Sarah Bailly, Vanessa Machault, Samuel Beneteau, Philippe Palany, Camille Fritzell, Romain Girod, Jean-Pierre Lacaux, Philippe Quénel, Claude Flamand

**Affiliations:** 1 Epidemiology Unit, Institut Pasteur in French Guiana, Cayenne 97306, French Guiana; bailly.sarah03@gmail.com (S.B.); samuel.beneteau@ntymail.com (S.B.); camillefritzell@gmail.com (C.F.); philippe.quenel@ehesp.fr (P.Q.); 2 Aerology Laboratory, Observatoire Midi-Pyrénées (OMP), Université Paul Sabatier, 31062 Toulouse, France; vanessamachault@yahoo.com.br (V.M.);; 3 Météo-France, Direction Antilles-Guyane, Fort-de-France 97262, Martinique; philippe.palany@gmail.com; 4 Medical Entomology Unit, Institut Pasteur in French Guiana, Cayenne 97306, French Guiana; romain.girod@pasteur.fr; 5 University Rennes, Inserm, EHESP, Irset (Institut de Recherche En Santé, Environnement et Travail)—UMR-S 1085, 35000 Rennes, France; 6 Epidemiology and Public Health Unit, Institut Pasteur in Cambodia, Phnom Penh 12201, Cambodia; 7 Mathematical Modelling of Infectious Diseases Unit, Institut Pasteur, UMR2000, CNRS, 75015 Paris, France

**Keywords:** dengue virus, *Aedes aegypti*, spatiotemporal modeling, remote sensing, vector control, French Guiana

## Abstract

French Guiana lacks a dedicated model for developing an early warning system tailored to its entomological contexts. We employed a spatiotemporal modeling approach to predict the risk of *Aedes aegypti* larvae presence in local households in French Guiana. The model integrated field data on larvae, environmental data obtained from very high-spatial-resolution Pleiades imagery, and meteorological data collected from September 2011 to February 2013 in an urban area of French Guiana. The identified environmental and meteorological factors were used to generate dynamic maps with high spatial and temporal resolution. The study collected larval data from 261 different surveyed houses, with each house being surveyed between one and three times. Of the observations, 41% were positive for the presence of *Aedes aegypti* larvae. We modeled the *Aedes* larvae risk within a radius of 50 to 200 m around houses using six explanatory variables and extrapolated the findings to other urban municipalities during the 2020 dengue epidemic in French Guiana. This study highlights the potential of spatiotemporal modeling approaches to predict and monitor the evolution of vector-borne disease transmission risk, representing a major opportunity to monitor the evolution of vector risk and provide valuable information for public health authorities.

## 1. Introduction

Arboviral diseases, transmitted by infected arthropods, have emerged as a significant public health concern [1,2,3,4]. French Guiana, an overseas French department with a population of 300,000 in Northeast South America, has faced multiple outbreaks of dengue, chikungunya, and Zika viruses in recent years, with *Ae. aegypti* being the sole known vector responsible for transmission in the country [5,6,7,8,9,10,11]. After a period of no dengue virus circulation between 2015 and 2018, dengue virus activity gradually intensified, culminating in a new epidemic in 2020 involving two serotypes [12,13], resulting in 12,300 clinical cases and four deaths [14].

While vaccines have been developed for some arboviral disease prevention, vector control remains the cornerstone of prevention efforts. In French Guiana, control efforts involve indoor and outdoor deltamethrin spraying to target adult mosquitoes in homes with human cases and within a 100 m radius. Additionally, mechanical and chemical methods are used to eliminate larval breeding sites [15,16]. However, these interventions can be limited by delayed responses and the challenge of accurately identifying contamination sites. Effective mitigation of high transmission risks necessitates the continuous collection of entomological and epidemiological data at the local level. Unfortunately, the intermittent collection of entomological data, due to the substantial resources required for continuous monitoring, often results in imprecise information, hindering the accurate identification of high-risk areas. Consequently, detailed risk maps are essential for pinpointing areas with a high probability of transmission from larval breeding sites, thereby enabling precision public health interventions to optimize control efforts. The continuous collection of fine-scale data, combined with epidemiological information, is crucial for enhancing the effectiveness of control measures and improving our understanding of vector-borne disease transmission. 

The correlation between spatial and temporal patterns of *Ae. aegypti* larval distribution and dengue risk has been extensively studied and debated over the decades [17,18]. While some studies have identified significant links [19,20], traditional larval indices, such as the Breteau Index and House Index, have shown inconsistent correlations with dengue transmission [18,21]. This inconsistency raises questions about their reliability as universal predictors of outbreaks, as they frequently fail to establish clear, quantifiable associations for predicting dengue epidemics. The variability in these correlations across different geographic regions and temporal contexts underscores the need for more precise and context-specific models. Such models are crucial for accurately forecasting dengue risk and enabling more effective and targeted public health interventions.

Weather and climate conditions such as rainfall, relative humidity, and temperature [5,6,22], as well as environmental conditions including vegetation indices, land use, and urbanization rate [23,24] play pivotal roles in the evolution of dengue epidemics. These conditions exhibit spatial and temporal heterogeneity. Remote sensing tools, as a valuable resource for collecting environmental and meteorological data with various spatial, spectral, and temporal resolutions, are employed to monitor these conditions at global, regional, and local scales. These tools, combined with different machine learning algorithms, are used to detect associations with entomological indices. For instance, in a study conducted in 2014 in Martinique, Machault et al. used logistic regression to model the presence of houses potentially hosting *Ae. aegypti* larvae [25]. The presence of larvae was significantly associated with maximum humidity and asphalt surface predictors. In Thailand, Sarfraz et al. used a decision tree to predict the probability of vector reproductive habitats using temperature, rainfall, human density, land cover, and elevation factors [26]. Others researchers have utilized time-series [27] or spatial analysis [28] to map abundances and explore correlations with reported cases. Previous studies have demonstrated that the effects of climatic parameters on dengue epidemic incidence can vary significantly from one site to another, depending on the local epidemiological context. Despite the importance of dengue [5,7,9,12,29,30] and the past epidemics transmitted by *Ae. aegypti* in French Guiana [8,31,32], there is currently no model designed to develop an early warning system tailored to the entomological context. This gap is particularly critical given the need for precise and timely interventions to control the spread of dengue. By utilizing historical data, we aim to demonstrate the potential of a straightforward predictive model to enhance surveillance, target high-risk zones, and improve intervention strategies for arbovirus transmission in French Guiana. To address this need, our study focuses on understanding and modeling the fine spatial and temporal dynamics of *Ae. aegypti* larvae presence based on meteorological and environmental factors. Our research is centered in Matoury, a municipality on the French Guiana coast near Cayenne, to capture the intricate interactions between climatic conditions and mosquito breeding patterns. Additionally, we applied this larval model to all houses in the municipalities of Cayenne, Rémire-Montjoly, and Matoury during the 2020–2021 dengue epidemic [33]. This extrapolation aimed to evaluate the model’s ability to identify areas at high risk of dengue transmission across different municipalities.

## 2. Materials and Methods

### 2.1. Study Area

French Guiana, located in South America, lies north of the equator along the Atlantic Ocean. The climate is equatorial, characterized by high temperature and humidity. Relative humidity rarely drops below 80% and average monthly temperatures remain around 27 °C throughout the year. Rainfall exhibits significant seasonal variation due to the influence of migration from the Intertropical Convergence Zone (ZCIT). The average annual rainfall is approximately 3 m, with the majority occurring during the rainy season from December to June. Conversely, the dry season, spanning from July to November, experiences lower levels of rainfall. There are considerable fluctuations in annual rainfall accumulation from year to year, influenced by El Niño. During El Niño periods, French Guiana experiences precipitation deficits, while La Niña events lead to higher annual rainfall accumulations [34,35]. Environmental conditions exhibit more heterogeneity across the territory compared to meteorological conditions. Ninety percent of the territory is covered by the Amazon rainforest, contributing to the region’s rich biodiversity. Most of the population resides along the coast, where the three main municipalities are located: Cayenne and its surroundings, Kourou, and Saint-Laurent-du-Maroni municipalities.

### 2.2. Data Collection

**Figure 1:**
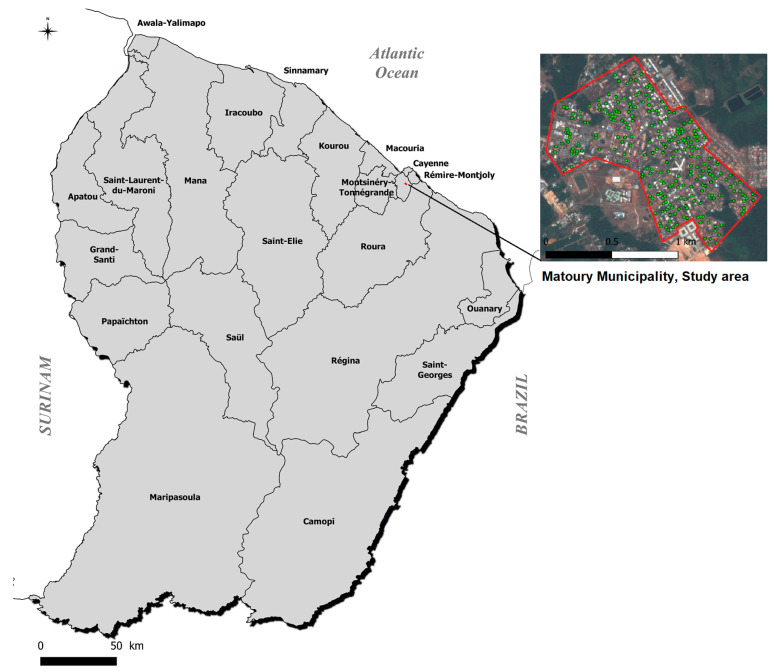
French Guiana, the studied area, and the center of Matoury. Source IGN—Open Street Map.

**Figure 2:**
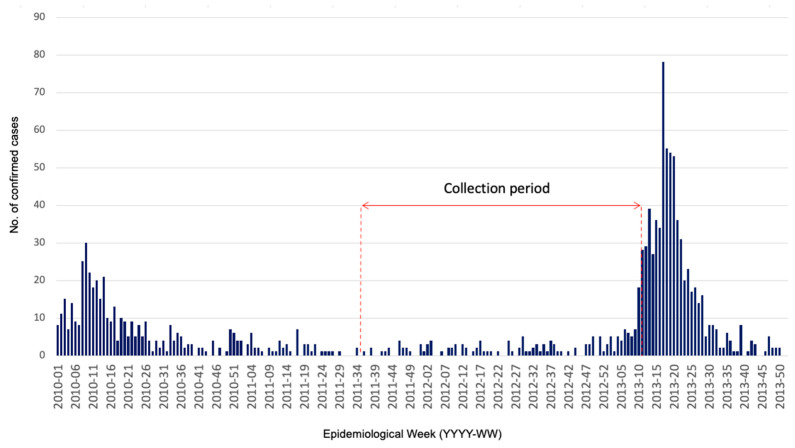
Number of confirmed cases collected by the regional system of epidemiological surveillance and entomological collection period in Matoury, French Guiana.

We conducted an entomological monitoring study over an 18-month period from September 2011 to February 2013 in the urban center of Matoury, (Figure 1), an adjacent municipality to the main city of Cayenne. At the time of the study, Matoury had a population of 29,712 inhabitants, covering an area of 137 km^2^, resulting in a population density of 217 inhabitants/km^2^. The town’s urbanization was heterogeneous, surrounded by dense forests, with the central area comprising 875 easily accessible houses, isolated from the rest of the town, representing urbanization in French Guiana. The monitoring period included 16 months of inter-epidemic phase and 2 months of dengue epidemics at the end of the study period (Figure 2). Each month, a 3-day monitoring session was conducted, during which we randomly selected twenty houses using a sampling plan with replacement. The objective was to identify and quantify the presence and types of water-holding containers, as well as to assess the presence or absence of larvae in both domestic and peridomestic containers.

During the study period, we collected daily meteorological data from the Météo France “Félix Eboué” station at Matoury airport including rainfall, minimum, average, and maximum temperatures, temperature amplitude, relative and absolute humidity, sea-level pressure, vapor pressure, global radiation, and sunshine duration, among others. For each trapping date, we averaged or cumulated 243 variables over different previous periods: 5, 10, 15, 20, 25, and 30 days, considering lag effects by integrating characteristics calculated over the last 5–10, 5–15, 5–20, 5–25, and 5–30 days. To enhance spatial rainfall information, we used satellite observations from the Tropical Rainfall Measuring Mission (TRMM). We obtained data from the nearest pixel to the area or the average of surrounding pixels.

The Pleiades satellite constellation provided high-resolution images (50 cm) with frequent updates, covering every point on the globe every three to four days. We acquired two pairs of optical Pleiades images, covering the dry season and the beginning of the rainy season, on 23 September 2012 and 10 December 2013, respectively. These images were projected in WGS 84, UTM Zone 22 N, and geometrically corrected using the SRTM 90 m database by NASA. We applied radiometric, atmospheric, and geometric corrections to the images, processed using ENVI 5.1 and ENVI EX (Exelis Visual Information Solutions).

We used QGIS version 2.12.3 with Orfeo Toolbox (OTB) to calculate 14 indices grouped into three categories, including vegetation (NDVI, TNDVI, RVI, SAVI, MSAVI2, GEMI, ARVI, AVI, IPVI), land use (RI, IB, IB2), and water (NDWI2, NDTI) based on spectral characteristics related to physical and biological features. We calculated atmospheric-effect indices (e.g., GEMI) before applying atmospheric correction to the images.

For land cover maps, we classified each image into eight classes: building, pool, asphalt, water, bare ground, light vegetation, green grass, and tree. We employed supervised maximum likelihood pixel classification in ENVI 5.1, using training polygons digitized by an operator based on spectral signatures of each class. Each pixel was assigned to the class with the highest probability based on these spectral signatures. The kappa coefficient, a measure of classification accuracy, was 0.93 for each image, indicating good agreement between the resulting classes and validation areas.

We performed object-oriented classification to enhance classification accuracy and avoid class confusion. This approach considers spectral information and spatial environment. We conducted segmentation and merging of objects based on rule operations in the ENVI FX module. Using confusion trees between pixel and object classifications, we better classified objects like blue or white roofs and swimming pools. For each house, identified by its centroid (from IGN cadastral data), we extracted various information from the immediate environment, calculating averages of all indices and determining land use class zones for buffer zones at 10, 20, 30, 40, 50, 100, 200, 300, 400, and 500 m around the house. We also calculated the distance to the first pixel of each class and the area of each land use class around the experimental unit.

We obtained epidemiological data of confirmed dengue cases, spanning from November 2019 to August 2020, within the Cayenne municipality and aggregated into 100 m × 100 m pixels, from the regional epidemiology unit of Santé Publique France as part of routine surveillance and epidemic management activities.

### 2.3. Statistical and Spatial Analyses

We compiled a large and diverse database, consolidating entomological data with 747 environmental and 195 meteorological factors. We employed univariate logistic regressions to model the probability of larvae presence, testing each of the 942 variables as explanatory factors. Variables with *p*-value < 0.25 were retained in the initial set of Boosted Regression Tree (BRT) variables, resulting in 317 explanatory variables. We conducted the analysis of larvae presence/absence using a BRT model, fitting a Bernoulli distribution with a flexible probability structure to characterize the relationship between the variable of interest and numerous explanatory variables. To refine the model and improve interpretability, we prioritized variables that had the most interpretable biological or physical significance and reduced those that were highly correlated with other variables in the model. This process ensured that the retained variables contributed unique and meaningful information to the model. We set specific parameters for the BRT model, including a tree complexity (tc) value of 5, a learning rate (lr) value of 0.001, and an initial number of trees (nt) of 50. Cross-validation was performed with a bag fraction of 0.5, using a portion of observations not used to build the model.

We measured the strength of association between each explanatory variable and the outcome using the ROC (Receiver Operating Characteristic) curve. We extrapolated the resulting model to the municipalities of Cayenne, Rémire-Montjoly, and Matoury during the last dengue epidemic period (from November 2019 to August 2020) to compare the locations of reported cases with the hotspots identified by the model.

We obtained a complete meteorological dataset without missing data from meteorological stations. We used a Pleiades image taken on 20 December 2017 for analysis. We correlated the predicted entomological data and epidemiological variables (geolocated biologically confirmed cases from November 2019 to August 2020) using different grid sizes, including 500 × 500 m and 1000 × 1000 m grids, as well as IRIS (homogeneous geographical and demographic micro-neighborhoods). We visualized these correlations using choropleth maps, representing the data by discretized ranges of values. We performed all statistical analyses using R software version 3.0.2.

## 3. Results

### 3.1. Overview of Larval Data and Modeling Approach

We collected larval data from 261 different houses, surveyed between one and three times, resulting in 333 observations, with 27 houses declining to participate across the various sessions. Among the participating houses, 41% were positive for *Ae. aegypti* larvae. We developed the final Boosted Regression Tree (BRT) model using six explanatory variables, narrowed down from an initial set of three hundred seventeen obtained via logistic regression. The model incorporated three environmental variables accounting for 59% of the cumulative influence: surface of low vegetation within a 100 m radius (26%), Mean Redness Index (RI) within a 50 m radius (18%), and Normalized Difference Tillage Index (NDTI) within a 200 m radius (15%). Additionally, three weather variables contributed to 41% of the model’s cumulative influence: mean temperature over the previous 5 days (19%), minimum temperature over the previous 25 days (12%), and rainfall over the previous 3 days (10%)

**Figure 3:**
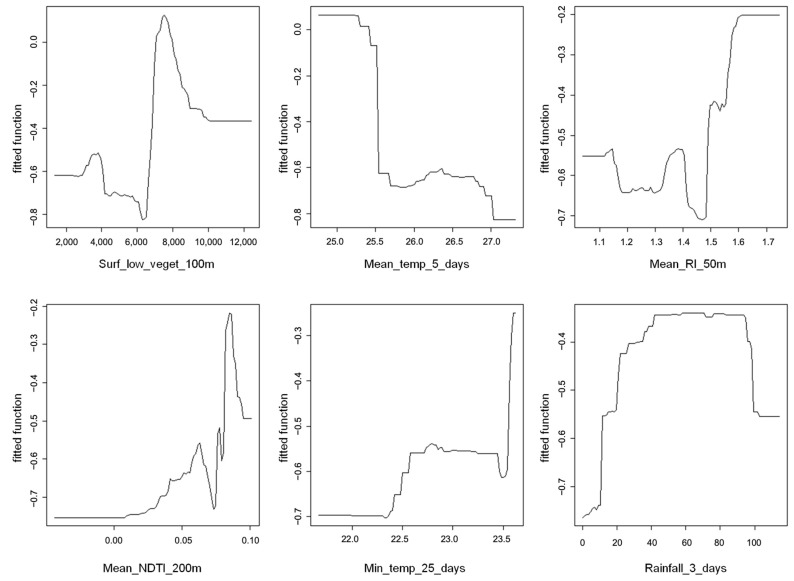
Graphs of associations between explanatory variables and the presence of larvae.

Figure 3 illustrates the directions of the associations and thresholds at which predictions change. For instance, a mean temperature of 25.5 °C over the previous 5 days decreases the probability of *Ae. aegypti* larvae presence. Conversely, for RI, beyond the cutoff point of 1.5, the probability of larvae presence increases. Cumulative rainfall over the previous 3 days constitutes a high-probability plateau for the presence of *Ae. aegypti* larvae, between 10 mm and 100 mm.

To account for the dynamic nature of mosquito populations and their environments, we added an entomological situation variable to the six other variables. This variable, representing the average probabilities of larvae presence in houses within a 30 m radius over the previous 7 days, was created using the initial model run and then incorporated into the second modeling run to predict larvae presence/absence for each geolocated house. The area under the ROC curve was 0.72.

### 3.2. Larvae Risk Mapping

**Figure 4:**
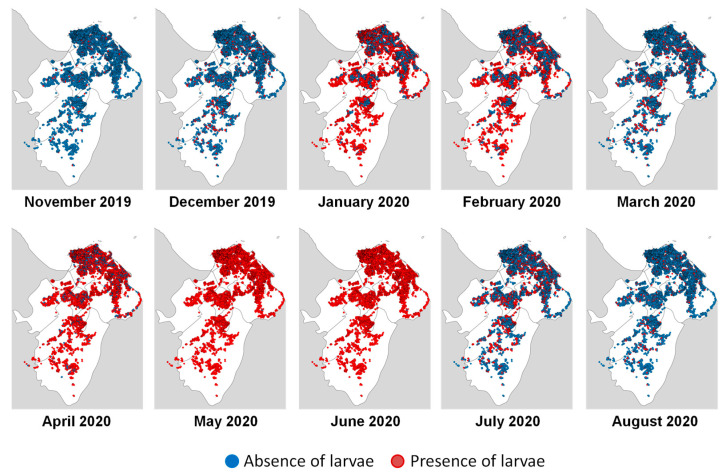
Monthly entomological risk maps from the modeling experiment based on data from November 2019 until August 2020 in the Cayenne area (Cayenne, Matoury, and Rémire-Montjoly municipalities).

During the 10 months of the studied epidemic period (2019–2020), the extrapolated model (Figure 4) assessed the risk of *Ae. aegypti* larvae presence (red) or absence (blue) for each house. No house was at risk every day of the study period, while all buildings appeared at risk for at least 100 days. The positivity rate varied between 3.8% and 99.6% (Table 1). The highest entomological risk occurred in May and June, with larvae presence positivity rates above 99%. Conversely, at the beginning and end of the epidemic period, the positivity rates were lowest, with November at 3.8% and August at 6.6%.

**Figure 5:**
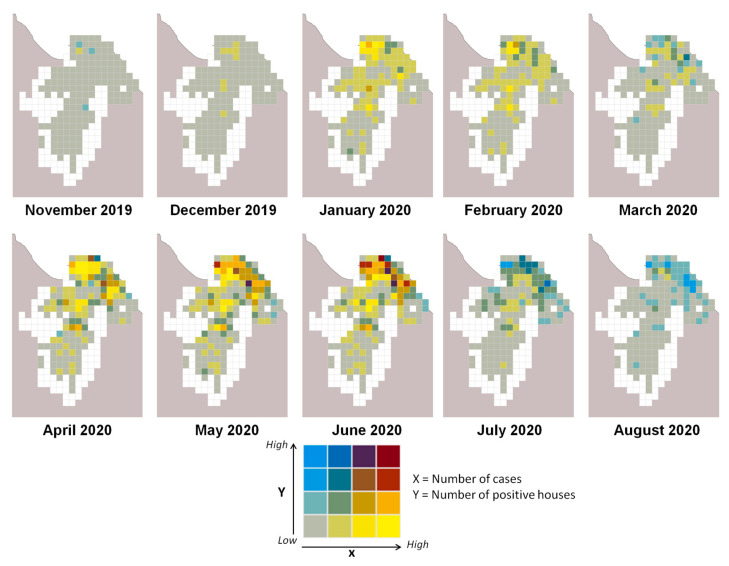
Correlation between the number of cases and the number of positive houses for the months where the model was extrapolated, Cayenne area (Cayenne, Matoury, and Rémire-Montjoly municipalities).

The highest positive correlations between entomological and epidemiological variables were found using a 1000 m × 1000 m grid, with correlations peaking between June and August 2020 (Table 1). Choropleth maps (Figure 5) revealed spatial heterogeneity in entomological and epidemiological results. In November and December 2019, at the epidemic’s onset, the number of cases and predicted positive houses was low, with no clear patterns emerging. As case numbers and predicted positive houses increased, distinct patterns appeared, with April, May, and June showing some grids as hotspot areas.

## 4. Discussion

We successfully utilized very high-resolution satellite imagery and daily meteorological field data to identify environmental variables that determine the presence of *Ae. aegypti* larvae. We investigated the close and more distant environment of the houses, considering radius areas of 50, 100, and 200 m. Interestingly, no larger-scale variables were found to be associated with larvae risk, indicating that the biological and physical phenomena leading to the presence of larvae occur primarily in close proximity to houses. During the “wet” season, we observed that the probability of larvae presence increased with the surface area of lightly vegetated land within a 100 m radius. This effect has been previously demonstrated in Martinique, where “sparsely vegetated soil” was identified as a risk factor for water lodges at the same scale [25]. Additionally, our results showed that land characteristics influenced vector risks within a 50 m radius, illustrating that the characteristics of the surroundings of a house, such as the yard or garden, impact the presence or absence of water deposits and the ability of larvae to develop in those deposits. The well-known relationship between land characteristics and vegetation can influence the presence of *Ae. aegypti* by providing shade for their roost [36]. Moreover, moist environments at low temperatures were found to favor larval development, while the supply of nutrients in water breeding sites attracts females for egg laying and larvae feeding [37], resulting in better survival rates and larger females [38,39]. A higher density of larvae in a location is evidently partly related to the presence of a high density of females. The NDTI in a 200 m buffer indicates bare rooftops and unvegetated surfaces, signifying urbanization. This index plays a positive role up to a threshold, as urbanization provides artificial lodgings and blood meal within this 200 m scale. The identification of NDTI as a significant predictor highlights the importance of urbanization in creating environments conducive to *Ae. aegypti* breeding [40,41]. By capturing the extent of bare surfaces and built-up areas, NDTI serves as a proxy for urban density, where anthropogenic activities increase the availability of breeding sites and suitable conditions for mosquito survival.

Furthermore, we identified three meteorological variables that remain constant in space and represent both the situation on previous days and the seasonal level. The average mean temperature in the preceding five days is a key factor that affects the likelihood of larvae presence, which is highest at around 25.5 °C. High air temperatures are not conducive to adult survival. The average minimum temperature in the preceding 25 days was positively associated with the presence of larvae, with two thresholds observed at approximately 22.5 and 23.5 °C, highlighting that cooler seasons are less favorable for vector presence. Additionally, the cumulative rainfall in the previous 3 days impacted the presence of larvae, quickly reaching a limit. When rainfall exceeded ten millimeters in three days, the probability of the presence of larvae reached its maximum. This finding is particularly relevant in our study area, where breeding sites such as cups, pots, and watering cans were small and quickly filled with water. Overall, the biological and physical significance of the identified predictors underscores their relevance in shaping the spatial and temporal dynamics of *Ae. aegypti* larval presence. These insights provide valuable guidance for targeted vector control strategies, especially in urbanized areas where intervention efforts can be concentrated based on predictive modeling outcomes.

The extrapolation of the model showed that March was less favorable for the presence of larvae than the other months of the rainy season. This month corresponds to the “short March summer”, when rainfall decreases significantly, between the short rainy season (January–February) and the long rainy season (April–June). As soon as the main rainy season starts, the probability of larval presence increases again. 

The spatiotemporal risk maps produced in this study are a significant contribution to the development of targeted operational control systems for arboviral diseases in urban areas. While it is widely accepted that entomological risk alone cannot fully predict the occurrence of human cases of arbovirus, as other factors such as socioeconomic level, virus circulation, and protective behaviors also play crucial roles, the presence of vectors remains an essential element in assessing the risk of transmission, especially when associated with virus circulation. Our proposed approach aims to identify areas at high risk of transmission by combining the presence of vector risk and the presence of the virus, as depicted in the choropleth maps. These maps can effectively direct vector control actions to hotspots areas. The core strength of our approach lies in its flexibility. While the specific parameters of the model (such as temperature thresholds and rainfall patterns) are influenced by the local climate of the study area, the overall framework is designed to be adaptable. By recalibrating the model with region-specific data, such as different temperature ranges, rainfall patterns, and vegetation cover, it can be applied to other tropical regions, including those with a tropical monsoon climate. However, there are limitations in the collected entomological data. For instance, we did not consider the productivity of breeding sites for larvae, and it might have been interesting to model only the presence of larvae at the most productive sites. Nevertheless, this may not be a significant issue in the specific case of French Guiana, as productivity was fairly dichotomous: close to 20% at positive sites with buckets or watering can containers and plants, versus close to 0% for all other types of sites. Another limitation of the study pertains to the methodology used to explore the associations between modeled entomological risk and case occurrence. While our study relied on the surveillance system for biologically confirmed cases, it is important to note that this system may not detect all dengue cases, potentially underestimating the actual number of cases in the control houses.

Looking ahead, it will be intriguing to identify geolocalized indicators that are more representative of epidemiological risk than individual confirmed cases, allowing us to better highlight the link between entomological risk and epidemiological risk. Future steps include implementing new strategies to produce high-resolution local maps across the country and integrating the proposed approach into the master plan of information systems dedicated to the surveillance of priority diseases in French Guiana, enabling precision public health actions to be deployed by control operators.

## Figures and Tables

**Table 1:** Correlation rate between entomological and epidemiological data.

	Number of Positive Cases	Number of Positive Houses	Proportion of Predicted Positive Houses (%)	Correlation Rate
November 2019	3	1125	3.8	0.2
December 2019	0	2339	7.9	-
January 2020	8	15,796	58.6	0.3
February 2020	11	10,730	38.6	0.2
March 2020	29	3707	9.0	0.2
April 2020	79	20,874	72.8	0.3
May 2020	121	25,610	99.6	0.4
June 2020	156	25,608	99.6	0.6
July 2020	168	6890	24.6	0.5
August 2020	122	2033	6.6	0.5

## Data Availability

The data used in this study are available upon request from the authors or the Institut Pasteur of French Guiana—Tel: +594(0)594292600; +594(0)594292618, Contact: https://www.pasteur-cayenne.fr/contact/, accessed on 1 August 2024).
